# Plant-Derived Alkaloids as a Potential Source of Treatment for Colorectal Cancer over the Past Five Years: A Comprehensive Review

**DOI:** 10.3390/plants13192723

**Published:** 2024-09-29

**Authors:** Tchangou Gaetan Tabakam, Tshepiso Jan Makhafola

**Affiliations:** Centre for Quality of Health and Living, Faculty of Health and Environmental Sciences, Central University of Technology, Bloemfontein 9300, Free State, South Africa

**Keywords:** alkaloids, axidimin C, axidimin D, chaetocochin J, colorectal cancer

## Abstract

The gastrointestinal cancer known as colorectal cancer (CRC) is caused by a variety of genetic and epigenetic alterations in the intestinal epithelium of the colon and rectum. It is becoming more common every year. In view of this significant progress, it is urgent and imperative for researchers to work more in this direction in order to improve this health situation that is a major concern for society. Certain phenomena, such as the development of resistance by certain cells as well as the failure of certain therapies, play a part in the significantly changed situation. However, plants have always been used for their therapeutic virtues due to the large number of compounds they contain. Among them, alkaloids (more than 20,000 alkaloids have been isolated from plants, of which about 600 are known to be bioactive), which are one of the most diverse and extensively investigated classes of compounds among natural products, can be consider as a promising approach with regard to their numerous biological activities in general and, in particular their activities against colorectal cancer. This work aims to undertake deeper research on the examination of alkaloids that can be used as lead compounds in the treatment of colorectal cancer. The databases used during the literature searches were Web of Science, PubMed/Medline, and Scopus. This methodology allowed us to obtain 11 studies and 24 alkaloids (axidimins A–D, tabersonine, 19*R*-hydroxytabersonine, 11-hydroxytabersonine, 11-methoxytabersonine, vandrikidine, fusiformine A, 3-oxotabersonine, 3-oxo-11-methoxytabersonine, melodinine W2, venalstonidine, scandine, (–)-larutienine A, solasonin, berbamine dihydrochloride, nitidine chloride, GB7 acetate, berberine, boldine, Worenine, and chaetocochin J). Axidimin C and axidimin D showed significant cytotoxic effects on CRC (HCT116 cells) with IC_50_ values of 5.3 and 3.9 μM, respectively, and they were more active than 5-fluorouracil and etoposide (IC_50_ = 6.4 and 10.6 μM, respectively) taken as references. These two compounds induced G_2_/M phase arrest in HCT116 cells by downregulating cyclin B1 and cdc2 expression. Subsequently, promoting apoptosis via modulation of Bax and Bcl-2 levels, they enhanced p38 MAPK expression, leading to G_2_/M cell cycle arrest and apoptosis in HCT116 cells. Chaetocochin J possess significant activity against three different CRC cell lines [RKO (0.5 μM < IC_50_ = 0.56 μM < 1.0 μM), HCT116 (0.5 μM < IC_50_ = 0.61 μM < 1.0 μM) and SW480 (0.5 μM < IC_50_ = 0.65 μM < 1.0 μM)]. The 21 remaining compounds have a moderate anti-colorectal cancer activity. Thus, we believe that axidimin C, axidimin D and chaetocochin J could be promising compounds to fight colorectal cancer cell carcinoma. Nevertheless, future analysis should be performed on the study of the toxicologies of axidimin C and axidimin D.

## 1. Introduction

Colorectal cancer (CRC) is a type of cancer that affects the colon (large intestine) or rectum, also called colon cancer. It is one of the most common types of cancer worldwide and can cause severe harm and death [1]. In 2020, approximately 1.9 million cases of CRC have occurred and at least 0.9 million of people have died because of this disease around the world [2,3,4,5]. Colorectal cancer (CRC) is a real and dangerous disease problem worldwide. It has been reported in the GLOBOCAN estimation provided by the International Agency for Research on Cancer (IARC) that this CRC is classified as the second most common cause of cancer-related death (after lung cancer) in the world, with an estimated number of 935,173 deaths in 2020 [5]. CRC is widespread around the world, particularly in Europe, the eastern Mediterranean region, Oceana, Asia, Latin America, North America, and Africa [3,5,6]. The GLOBOCAN estimates suggest a higher contamination rate for CRC in men than women, with 515,637 and 419,536 deaths, respectively [3,5,6].

In view of the rapid evolution of the mortality rate caused by CRC, efforts must be multiplied in the search for solutions. Globally, the impact and mortality of CRC is growing year over year, and there could be more than 2.2 million new cases and 1.1 million deaths by 2030 [7,8]. This impact and mortality of CRC vary significantly, depending on factors such as gender, age and region, and socio-economic development [9]. In many developing countries, morbidity and mortality rates are increasing exponentially [7].

The treatment of colorectal cancer has undergone several advances, such as radiotherapy, surgery and chemotherapy, including 5-fluorouracil or irinotecan, intervening significantly in the management of CRC diagnosed at high levels. However, several problems are still encountered during these treatments, such as toxicity of side effects as well as therapeutic failures associated with small-molecule drugs and the rapid loss of efficacy of monoclonal antibody therapies [10]. The resistance of some colorectal cancer cell lines is also an issue to consider. Faced with these limitations, it is urgent and imperative to research other healthier and more effective methods in the treatment of CRC. It is widely known that through the ages, people took advantage of nature in order to meet their primary needs. This also applies to the use of natural products as medication for a wide range of diseases encompassing cancer [11]. Research on plants around the world has led to discovered of several classes of compounds, including alkaloids, with good biological activities on colorectal cancer (CRC).

The first known source of alkaloid compounds was plants: “Alkaloids are organic substances containing nitrogen of natural origin with a greater or lesser degree of basic character”. Alkaloids encompass a huge class of about 12,000 natural products [12]. Alkaloids are one of the most diverse and extensively investigated classes of compounds among natural products [13]. Alkaloids have been shown to possess many biological activities, including anticancer properties. For example, berberine is an alkaloid that has demonstrated a notable impact during the development of colorectal cancer formation [14]. We hope that other compounds in the same family will have much more pronounced activities.

According to the above-mentioned information regarding the rapid evolution of this type of cancer (colorectal cancer), we decided to use the tools and the different methods described in the systematic review protocols (PRISMA) to firstly, identify all the alkaloids isolated from plants with activity against colorectal cancer during the past five years; secondly, to identify among these compounds those that have shown significant activities; and finally to fix the attention of researchers on the promising ones in order to undertake additional tests, if necessary, and use them as lead compounds in the treatment of colorectal cancer.

## 2. Results and Discussion

### 2.1. Characteristics of Results from Literature Search

**Figure 1:**
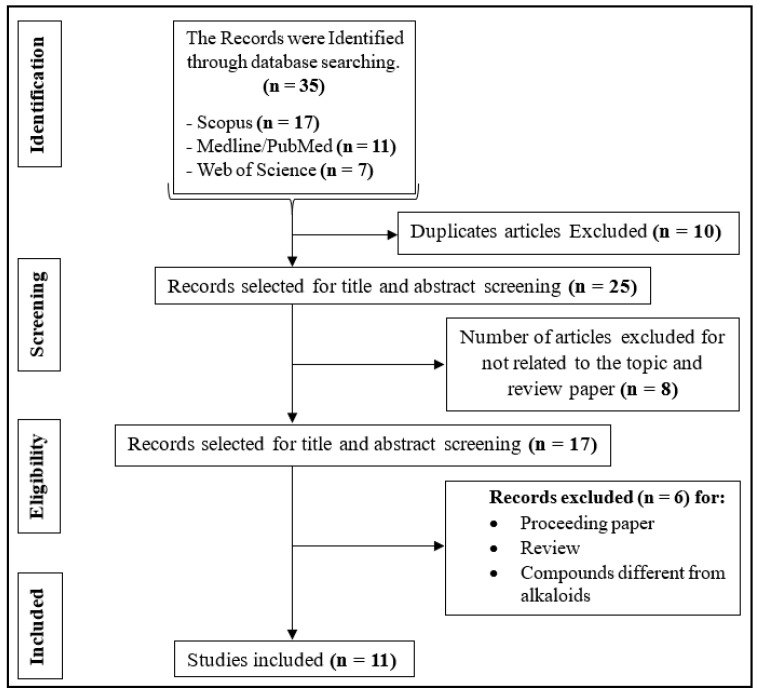
Schematic flow diagram for the selection of study.

**Figure 2:**
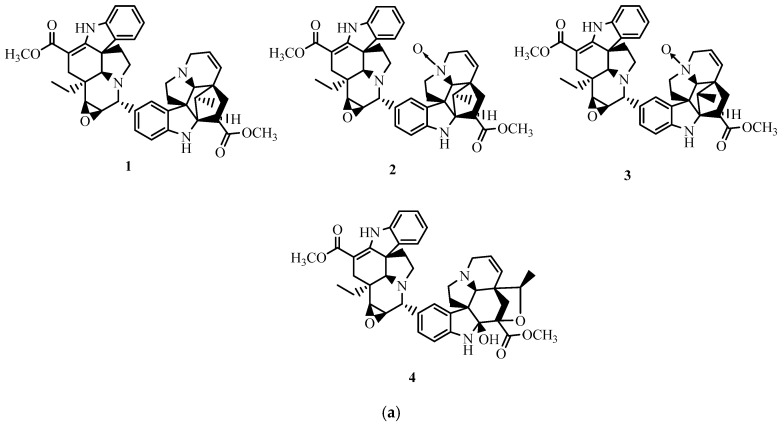

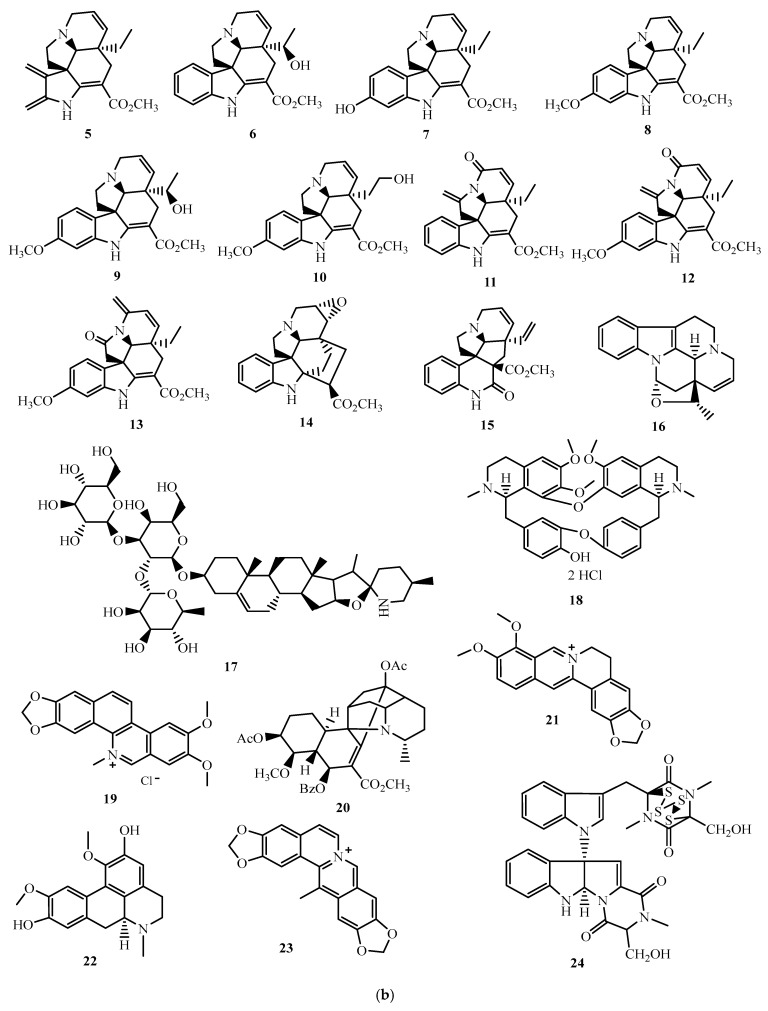
(**a**) Structure of new isolated bioactive alkaloids. (**b**) structure of known isolated bioactive alkaloids.

The literature search allowed us to find 11 results regarding the effects of natural alkaloids against colorectal cancer (Figure 1). A total of 24 compounds belonging to different sub-classes of alkaloids were revealed by the studies, enumerated below: axidimins A–D (**1**–**4**) [15], tabersonine (**5**), 19*R*-hydroxytabersonine (**6**), 11-hydroxytabersonine (**7**), 11-methoxytabersonine (**8**), vandrikidine (**9**), fusiformine A (**10**), 3-oxotabersonine (**11**), 3-oxo-11-methoxytabersonine (**12**), melodinine W2 (**13**), venalstonidine (**14**), scandine (**15**), (–)-larutienine A (**16**) [15], solasonin (**17**) [16], berbamine dihydrochloride (**18**) [17], nitidine chloride (**19**) [18], GB7 acetate (**20**) [19], berberine (**21**) [20,21], boldine (**22**) [21], worenine (**23**) [22], and chaetocochin J (**24**) [23] (Table 1, Figure 2). All studies have documented in vitro activity. Two of them did, however, also report in vivo activities [18,20]. All the research was conducted in China, with the exception of the study of [21], which was conducted in Malaysia.

### 2.2. Axidimins C and D

Axidimins C (**3**) and D (**4**) are monoterpenoid indole alkaloid dimers isolated from *Melodinus axillari*s *(Melodinus)*, comminuted herbs belonging to the Apocynaceae family [15]. These two compounds exhibited a significant cytotoxic effect on HCT116 cells with IC_50_ values of 5.3 and 3.9 μM, respectively, outperforming 5-fluorouracil and etoposide (IC_50_ = 6.4 and 10.6 μM, respectively) under similar conditions, according to the cut-off point of [24]. Using flow cytometry and Western blot analysis, it was discovered that axidimin C and D downregulated the expression of cyclin B1 and cdc2 in HCT116 cells, inducing G2/M phase arrest. This, in turn, promoted apoptosis by altering the levels of Bax and Bcl-2. Axidimins C and D increased p38 MAPK expression, which caused G2/M cell cycle arrest and death in HCT116 cells [15]. In view of these multiple activities, these two compounds display a large spectrum and could be used in combination or not against CRC.

### 2.3. Axidimins A and B

Axidimins A (**1**) and B (**2**) were isolated from the same source as axidimins C (**3**) and D (**4**) [15]. They presented a moderated activity on HCT116 cells with IC_50_ values of 12.0 and 15.7 μM, respectively. Further studies should be undertaken on them in order to see if their combination with other molecules or their chemical transformation could potentialize their activity against cancer. It is therefore a great starting point for a new line of research.

### 2.4. Tabersonine

Tabersonine (**5**) is an alkaloid ester, a monoterpenoid indole alkaloid mainly isolated from the medicinal plant *Catharanthus roseus* (Apocynaceae) [25]. It is also isolated from *Melodinus axillari*s (Apocynaceae) [15]. This compound has remarkable biological properties that have led to their medical uses for a variety of human diseases; its cytotoxic effect was found to be outstanding. Its IC_50_ value on ten different types of human cancer cell lines ranged from 4.8 ± 0.4 mu g/mL to 22.5 ± 1.4 mu g/mL [26]. Tabersonine showed a moderated activity on HCT116 cells (IC_50_ = 27.2 μM) [15]. Based on these findings, further investigation of this compound could be good for future research on colorectal cancer.

### 2.5. 19R-Hydroxytabersonine and 11-Hydroxytabersonine

19*R*-hydroxytabersonine (**6**) and 11-hydroxytabersonine (**7**) belong to the monoterpenoid indole alkaloids group and are rare alkaloids mainly isolated from the Melodinus genus, such as *Melodinus suaveolens*, a plant of the Apocynaceae family [27]. They have also been isolated from *Melodinus axillaris* (Apocynaceae) and demonstrated a moderate activity against colorectal cancer HCT116 cells with an IC_50_ of 31.4 and 19.2 μM, respectively [15]. These few results on anticancer activity of these compounds could be a good motivation to carry out further research on them.

**Table 1:** Different modes of action into the biological cells and the anticancer mode of actions of some alkaloids enumerated in this work.

Compounds	Targets	Cell Lines	Mode of Action	References
Acidimins C (**3**) and D (**4**)	Cyclin B1 and cdc2	HCT116	Downregulation inducing G2/M phase arrest.	[15]
Acidimins C (**3**) and D (**4**)	Bax and Bcl-2	HCT116	Alteration promoting apoptosis.	[15]
Acidimins C (**3**) and D (**4**)	P38 MAPK	HCT116	Increase that caused G2/M cell cycle arrest and death.	[15]
11-methoxytabersonine (**12**)	AMPK/mTOR and JNK	H157	Activation caused autophagy.	[28]
Solasonin (**17**)	HDAC/P53/P21	SW620	Downregulation of HDAC. Increase P53 acetylation, increase P51 suppressing the growth of colorectal cancer.	[16]
Berbamine (**21**)	P21/Cyclin E1/Cyclin E2/CDK6/Cyclin D1/	HT-29, HCT-116, RKO, SW480	Increase P21 levels causing G1–S phase arrest; decreased cyclin E1, E2, D1 and CDK6.	[17]
Berbamine (**21**)	RT-qPCR		Downregulation on AKT1, EGFR, PDG-FR*α* and FGFR4.	[17]
Nitidine chloride (**19**)	RKO	HCT116	Suppress the cell proliferation.	[18]
Nitidine chloride (**19**)		HT29	Colony inhibition.	[19]
GB7 Acetate (**20**)	AMPK signaling pathway	HCT116	Suppression of cell proliferation.	[19]
GB7 Acetate (**20**)	AMPK signaling pathway	HCT116	Anti-metastatic, anti-metabolite capabilities.	[19]

### 2.6. 11-Methoxytabersonine

Isolated from some plant species, such as *Melodinus axillari*s (Apocynaceae), 11-methoxytabersonine (**8**), which is an aspidosperma-type alkaloid, showed a moderate activity against colorectal cancer on HCT116, with an IC_50_ value of 25.3 μM [15]. In addition, several studies demonstrated the important activities of this compound against several other types of cancer [28]. isolated the same compound from *Tabernaemontana bovina* (Apocynaceae) and noted its significant inhibition of the viability of human lung cancer cell lines H157 and A549. In fact, 11-methoxytabersonine (**8**) killed lung cancer cells via the induction of necroptosis in an apoptosis-independent manner [28]. It has also been demonstrated that 11-methoxytabersonine (**8**) strongly induced autophagy in H157 and A549, which played a protective role against 11-methoxytabersonine-induced necroptosis. Finally, the autophagy caused by 11-methoxytabersonine (**8**) was found to be via activation of the AMP-activated protein kinase (AMPK)/mammalian target of rapamycin (mTOR) and the c-Jun N-terminal kinase (JNK) signaling pathways in both cells (H157 and A549) [28]. Cell viability was markedly reduced by 11-methoxytabersonine (**8**). While observations in the literature indicate that 11-methoxytabersonine (**8**) had significant cytotoxic effects on a variety of cancer cell lines, the specific molecular processes involved are yet unknown. This could be a new subject for future research.

### 2.7. Vandrikidine

Vandrikidine (**9**) is a monoterpenoid indole alkaloid, which has been isolated from the plant *Catharanthus roseus* (Apocynaceae). According to the literature, vandrikidine has been shown to be effective against bacteria and displayed cytotoxicity against A549 lung cancer cells, and the IC_50_ values ranged from 5.6 to 77.1 *μ*M [19]. This compound also exhibited a moderate activity against colorectal cancer HCT116 cells with an IC_50_ value of 44.8 μM [15]. In view of all this information, there is a need for further research to explore its potential further as a therapeutic agent against colorectal cancer.

### 2.8. Fusiformine A

The monoterpenoid indole alkaloid fusiformine A (**10**) was isolated for the first time from *Melodinus fusiformis* (Apocynaceae), and its cytotoxic activity evaluated against the growth of human tumor cell lines (HL-60 and A-549) showed moderate cell growth inhibitory activity with IC_50_ values of 9.80 and 12.38 μM, respectively [29]. Furthermore, it was also isolated from *Melodinus axillari*s (Apocynaceae) and showed a moderated anti-colorectal (HCT116) cancer activity with an IC_50_ of 38.5 μM [15]. This compound must be investigated further on other colorectal cancer cells in view of its moderated activity on HCT116 cells.

### 2.9. 3-Oxotabersonine

In addition to the moderated activity of 3-oxotabersonine (**11**) against colorectal cancer HCT116 cells with an IC_50_ of 22.6 μM, this compound, which is a monoterpenoid indole alkaloid isolated from *Melodinus axillaris* (Apocynaceae) [15], has also been isolated from the seeds of *Voacanga africana* (Apocynaceae), exhibiting different inhibition effects on cancer cell lines with the IC_50_ values ranging from 4.8 μg/mL to over 100.0 μg/mL [25].

### 2.10. Venalstonidine

*Venalstonidine* (**14**) is a monoterpenoid indole alkaloid isolated mainly from Melodinus genus such as *Melodinus reticulatus* [30], while *Melodinus axillari*s belonging to the Apocynaceae family exhibited a moderated activity on colorectal cancer HCT116 with an IC_50_ of 47.7 μM [15].

### 2.11. (–)-Larutienine

(–)-*larutienine* (**16**) is a monoterpenoid indole alkaloid and has already been isolated from *Kopsia pauciflora* (Apocynaceae). It also did not show appreciable cytotoxicity towards KB cells with an IC_50_ > 30 μg/mL. It was also isolated from *Melodinus axillari*s (Apocynaceae) and showed moderate activity against HCT116 colorectal cancer cells, with an IC_50_ value of 26.1 μM [15].

### 2.12. Solasonin

In a concentration-dependent manner, solasonin (**17**), isolated from *Solanum nigrum* L. (Solanaceae), markedly suppressed the proliferation of CRC cells. The IC_50_ values of solasonin on SW620, SW480, and MGC803 cells were, respectively, 35.52, 44.1, and 46.72 μM. Treatment of the SW620 cell line with solasonin led to a significant downregulation of HDAC, an increase in P53 acetylation, and an increase in P21 [16]. The in vivo validation results demonstrated its ability to suppress the growth of colorectal cancer (CRC) that was linked to the downregulation of HDAC successfully. Increased apoptosis and P21-induced cycle arrest were caused by the increased acetylation of P53 caused by HDAC inhibition [16]. This result shows that this compound solasonin can be a good candidate for CRC.

### 2.13. Berbamine

Berbamine (**18**) is a bisbenzylisoquinoline alkaloid that has been found from the traditional herbal medicine *Berberis amurensis* Rupr. (Berberidaceae) [31]. It has been demonstrated that berbamine could inhibit CRC cell line growth and presented an inhibitory effect on the ability of migration and invasion in CRC cells. Additionally, BBM showed an inhibitory effect on CRC cells’ ability to migrate and invade. Berbamine increased p21 levels and caused the G1–S phase arrest, but it also decreased CyclinE1, CyclinE2, CDK6, and CyclinD1. The down-regulation of berbamine on AKT1, EGFR, PDGFRα, and FGFR4 genes was demonstrated by RT-qPCR [17]. For HT-29, HCT116, RKO, and SW480 cells, the IC_50_ values of berbamine at 24 h were 11.92, 14.51, 15.96, and 39.54 μM, respectively. It is also determined that berbamine may control the variation in ROS levels, which in turn controlled the growth and death of colon cancer cells. Berbamine’s ability to reduce colon tumor growth significantly in vivo was shown. Berbamine has the ability to cause G1-phase arrest in colorectal cancer cells while also causing S-phase arrest in cancer cells [17].

### 2.14. Nitidine Chloride

Nitidine chloride (**19**) is a benzophenanthridine alkaloid that can be found in *Zanthoxylum nitidum* (Roxb.) DC (Rutaceae). This compound inhibited the proliferation of RKO (2 μM), HCT116 (3.5 μM), and HT29 (6 μM) cells and also has the ability to strongly suppress cell proliferation at high concentrations; its significant colony inhibition of HT29 cells has been demonstrated [18]. Nitidine chloride (**19**) inhibits the growth of tumor cells and promotes apoptosis in tumor tissues by causing CRC cells to undergo apoptosis [18].

### 2.15. GB7 Acetate

This compound belonging to the galbulimima alkaloid family could be found in the plant species *Galbulimima belgraveana* (Himantandraceae). This compound showed activity that suppressed the proliferation and colony-forming ability of CRC (HCT116) cells, with an IC_50_ value of 97.75 μg/mL. In addition, GB7 acetate (**20**) is shown to have pro-autophagic capabilities via the AMPK signaling pathway in addition to anti-metastatic, and anti-metabolite capabilities in HCT 116 cells [19].

### 2.16. Berberine

Berberine (**21**) is a protoberberine alkaloid isolated from plants species. It was isolated from *Berberis* spp. (Berberidaceae) and *Tinospora* spp. (Menispermaceae). It demonstrated a moderate activity against HCT116 (10.30 μg/mL) [21] and from *Coptidis rhizoma* (Ranunculaceae) with the in vitro activity against HCT116 and SW480 [20].

### 2.17. Boldine

The aporphine alkaloid boldine (**22**) isolated from *Peumus boldus* (Monimiaceae) showed a moderated activity against HCT116 (IC_50_ = 37.87 μg/mL) [21].

### 2.18. Worenine

It has been demonstrated that the isoquinoline alkaloid worenine (**23**), isolated from *Coptis chinensis* (Renonculaceae), inhibited colorectal cancer cell growth [CRC (HCT116 and SW620; 18.30 and 15.19 μM, respectively.)], proliferation, cell cycle progression, and the Warburg effect by targeting HIF-1*α* in vitro [22].

### 2.19. Chaetocochin J

Chaetocochin J (**24**) is an epipolythiodioxopiperazine alkaloid first isolated from the secondary metabolites of *Chaetomium* sp. (Chaetomiaceae). This class of alkaloid has a large spectrum of biological activities. This compound was evaluated for its anti-CRC activity and the result showed that it had a strong proliferation inhibition effect with the IC_50_ value to CRC cells around 0.5 μM. In addition, chaetocochin J (**24**) also induces apoptosis of CRC cells in a dose-dependent manner with a stronger effect than topotecan. It has further been demonstrated that chaetocochin J exerts its anti-CRC function via AMPK and PI3K/AKT/mTOR pathways and further regulation of their downstream signaling cascade in CRC cells, including apoptosis and autophagy. These data potently suggest that chaetocochin J may be a potential drug candidate for CRC treatment [23].

### 2.20. Scandine, Melodinine W2 and 3-oxo-11-Methoxytabersonine

Scandine (**15**), melodinine W2 (**13**) and 3-oxo-11-methoxytabersonine (**12**) are all the monoterpenoid indole alkaloids found in *Melodinus axillaris* (Apocynaceae). They have demonstrated their moderated anti-colorectal cancer (HCT116) activity with IC_50_ = 42.9, 37.7, and 24.4 μM, respectively [15].

Table 1 shows the different mode of action into the biological cells and the anticancer mode of actions of some alkaloids enumerated in this work.

**Figure 3:**
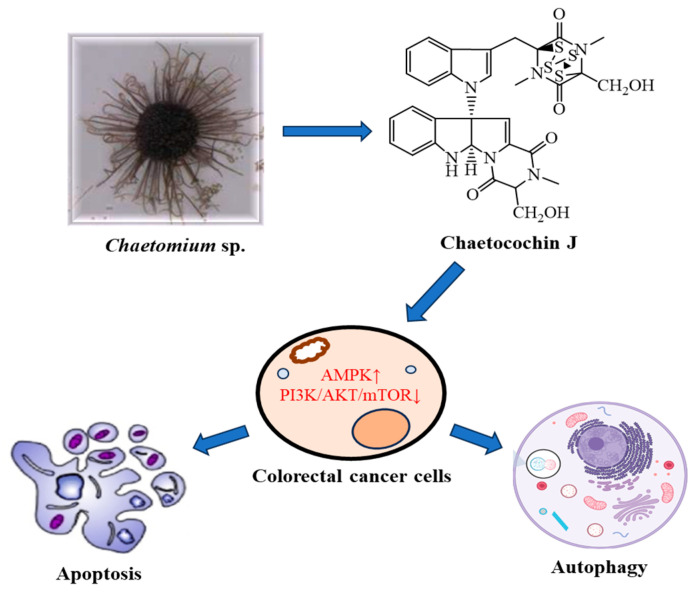
Mode of action of chaetocochin J (**24**) on colorectal cancer cells.

Figure 3 below illustrates the mode of action of chaetocochin J (**24**) on colorectal cancer cells.

Table 2 below shows the database [molecular formula and molecular mass (Cal.)] of all the alkaloids mentioned in the document.

## 3. Methods

### 3.1. Eligibility Criteria

The authors (T.G.T. and T.J.M.) each extracted data independently from all selected articles. Their attention focused on information concerning the study design, main results, and general mechanism of action. All geographic areas have been explored. Studies highlighting the activities of groups of compounds other than alkaloids were not considered; only the biological activities of alkaloids against colorectal cancer cell lines should explicitly be listed in this document. All results that have been published in the form of reviews, letters, editorials, conference abstracts, anonymous reports, unpublished works, commentaries, and criticisms have not been taken into account in this document. All in vitro/in vivo experimental studies based on the evaluations of the activities of alkaloids isolated from natural sources on colorectal cancer as the primary or secondary objective were selected eligible for our study. Table 3 contains the selected results and their characteristics, including the different alkaloids, their different classes, and the different types of colorectal cancer cell lines.

### 3.2. Data Sources with Search Tip

Google Scholar was used only to see if any publications existed that had not come up in the search using the other databases. A total of 665 abstracts were suggested for this purpose, but nothing was kept after thorough screening. Databases such as Scopus, PubMed/Medline, and Web of Science were consulted following a very specific methodology in the development of this work. From these databases, scientific work between 2018 and 2024 related to our subject was brought together. Our documentation was carried out in compliance with pre-established PRISMA standards [32]. There were no linguistic limitations. The search terms used in these four databases were “bioactive alkaloids” OR “plant-derived alkaloids” OR “isolated alkaloids” AND “colorectal cancer” OR “colon cancer” OR “colorectal tumor” OR “colorectal malignancy” OR “colorectal squamous cell”.

### 3.3. Study Choice

After the identified studies had been transferred to EndNote, duplicates were eliminated [32], and research abstracts and titles were created. During the second independent selection procedure, publications with titles and abstracts that satisfied the eligibility conditions were carefully reviewed in their entirety. Ultimately, the authors meticulously cross-checked the outcomes of each individual selection to determine the ultimate list of studies that would be incorporated into the study. The steps described in the PRISMA flow chart [32,33] were followed, and the documents were eliminated gradually until the total number of articles was obtained (Figure 1).

### 3.4. Data Gathering and Evaluation of Methodological Quality

In order to collect good data and perform a good evaluation, we proceeded as follows: the number of each alkaloid, the name and class of alkaloids, the plant source (family), the year, the country, the type of cancer cell line as well as the references (Surname of the first author) that was employed were all extracted. Each author extracted data independently. We decided to use a synoptic table to display the results (Table 3) as well as (Figure 2) to establish the chemical structures of these alkaloids.

## 4. General Discussion

Colorectal cancer is a dangerous cancer that is harmful to human health. In this study, we found that the data collected showed that almost all the plants studied are from China, and the work was carried out in China, with the exception of one study performed in Malaysia. The alkaloids listed mostly belong to the subclass of indole monoterpenoids [34]. We also found that the plants from which these alkaloids were isolated belong to those of the Apocynaceae family, with 16 out of 25 belonging to said family.

Much recent work demonstrating the anticancer potential of indole-containing alkaloids has been found [35]. Indole alkaloids are natural products extensively found in nature and have been proven to possess various pharmacological activities [35]. In recent years, pharmacological studies have demonstrated another potential of indole alkaloids, autophagy regulation [35]. The regulation may contribute to the efficacy of indole alkaloids in preventing and treating cancer [35]. Several indole alkaloids have shown an antiproliferative effect due to different mechanisms, namely by inducing apoptosis or arresting the cell cycle, in diverse cancer cell lines, including multidrug-resistant phenotypes [36]. Apocynaceae is a large family of tropical trees, shrubs, and vines with most species producing white latex. Alkaloids are part of the major metabolites of species [37].

The results of our study confirm the above-mentioned information regarding the anticancer properties of indole-alkaloids as well as the Apocynaceae family. Thus, previous studies demonstrated the anticancer property of indole-containing alkaloids as well as the Apocynaceae family. This allowed us to understand the anti-colorectal cancer activity of the selected indole alkaloids presented in this review.

Biological tests against colorectal cancer stem cells have been performed in vitro and may have been performed in vivo. In addition, the most-used strains have been those of HCT116. These tests should be performed on several other cell lines. However, we believe that the results are satisfactory on the HCT116 strains on which these compounds have previously been tested. The majority are an indication of the potential of these alkaloids, which can be exploited to fight colorectal cancer.

## 5. Limitation

This work was carried out under conditions in which the toxicology of many of the tested compounds has not been evaluated. This missing information is one of the limitations of this work. This work should be continued urgently by researchers with the aim of determining the degree of safety of these compounds. Another fundamental limitation is the lack of information or in vitro work performed with these compounds, because we do not know what form a drug can take (active, inert, less active, or transformation into a dangerous form) during its transition into a living organism. In the future, this should also be explored by researchers in order to gain complete information (in vitro and in vivo data) on these tested compounds.

## 6. Critical Synthesis and Perspectives

Regarding our development in the general discussion as well as the limitations that we have exposed, we have also noticed that the work has not been performed in the sense of highlighting the nanotechnological applications of these indole-containing bioactive alkaloids. However, currently it is known that noble metal nanoparticles like Ag, Au, Pd, and Pt have a variety of uses in different domains such as material science, medicine, and chemistry [38].

Because of their unique structural, catalytic, and optical characteristics as well as their enormous surface area, platinum nanoparticles have drawn a lot more interest from researchers, making them a viable option for biological and catalytic applications [39]. It has been reported that platinum nanoparticles are a competent and efficient drug carrier. Additionally, platinum nanoparticles have a wide range of medicinal uses, including antifungal, antibacterial, anti-diabetic and anticancer effects [39].

Due to the rarity of chemical medications that are therapeutically useful long-term [40], we suggest to researchers, as one of the perspectives of this work, to highlight the manufacture of platinum nanoparticles through the promising bioactive indole-containing alkaloids noticed in this work, such as axidimin C, axidimin D, and chaetocochin J.

## 7. Conclusions

This review summarizes all plant-derived alkaloids in their activity against colorectal cancer during the past five years. Currently, despite the existence of several existing cancer therapies, the rate of colorectal cancer is still expanding. Therefore, it is important and urgent to update the data on active compounds in general and alkaloids as in our particular case, which can be used as lead compounds in the development of new drugs to fight colorectal cancer. The results obtained from this work allowed us to conclude that among the 24 alkaloids collected during this review, axidimin C, axidimin D, and chaetocochin J were the most potent against colorectal cancer cell carcinoma. Chaetocochin J demonstrated significant activity against three different CRC cell lines (RKO, HCT116, and SW480). It could be more beneficial for pharmaceutical companies to produce bioactive drugs using the above-mentioned compounds in order to reduce the negative effect of colorectal cancer in humanity. Despite the lack of toxicology studies on human normal cells of these three promising bioactive alkaloids, these are very promising compounds for the future of colorectal cancer.

## Figures and Tables

**Table 2:** Database of bioactive isolated alkaloids.

Names of Compounds	Molecular Formula	Molecular Mass (Cal.)
***Monoterpenoid indole alkaloid dimers***		
Axidimin A (**1**)	C_42_H_46_N_4_O_5_	686.3468
Axidimin B (**2**)	C_42_H_46_N_4_O_6_	702.3417
Axidimin C (**3**)	C_42_H_46_N_4_O_6_	702.3417
Axidimin D (**4**)	C_42_H_46_N_4_O_7_	718.3366
***Monoterpenoid indole alkaloid***		
Tabersonine (**5**)	C_19_H_24_N_2_O_2_	312.1838
19*R*-Hydroxytabersonine (**6**)	C_21_H_24_N_2_O_3_	352.1787
11-Hydroxytabersonine (**7**)	C_21_H_24_N_2_O_3_	352.1787
11-Methoxytabersonine (**8**)	C_22_H_26_N_2_O_3_	366.1943
Vandrikidine (**9**)	C_22_H_26_N_2_O_4_	382.1893
Fusiformine A (**10**)	C_22_H_26_N_2_O_4_	382.1893
3-oxotabersonine (**11**)	C_22_H_22_N_2_O_3_	362.1630
3-oxo-11-methoxytabersonine (**12**)	C_23_H_24_N_2_O_4_	392.1736
Melodinine W2 (**13**)	C_23_H_24_N_2_O_4_	392.1736
Venalstonidine (**14**)	C_21_H_24_N_2_O_3_	352.1787
Scandine (**15**)	C_21_H_22_N_2_O_3_	350.1630
(–)-larutienine A (**16**)	C_19_H_20_N_2_O	292.1576
***Steroidal alkaloid***		
Solasonin (**17**)	C_45_H_73_N_2_O_3_	883.4929
***Bisbenzylisoquinoline alkaloid***		
Berbamine dihydrochloride (**18**)	C_37_H_40_N_2_O_6_	608.2886
Nitidine chloride (**19**)	C_21_H_18_ClNO_4_	383.0924
***Galbulimima alkaloid***		
GB7 acetate (**20**)	C_34_H_41_NO_9_	607.2781
***Protoberberine alkaloid***		
Berberine (**21**)	C_20_H_18_NO_4_^+^	336.1230
***Aporphine alkaloid***		
Boldine (**22**)	C_19_H_21_NO_4_	327.1471
***Isoquinoline alkaloid***		
Worenine (**23**)	C_20_H_14_NO_4_^+^	332.0917
***Epipolythiodioxopiperazine alkaloid***		
Chaetocochin J (**24)**	C_31_H_30_N_6_O_6_S_4_	710.1110

**Table 3:** Characteristics of studies included. (**a**) New alkaloids with anti-colorectal cancer properties within the last five years. Characteristics of studies included. (**b**) Known alkaloids with anti-colorectal cancer properties within the last five years.

**(a)**						
**Alkaloids** **(Number)**	**Classes**	**Plant Source (Family)**	**Year**	**Country**	**Type of Cancer Cells Lines**	**References (Name of First Author)**
Axidimin A (**1**)	Monoterpenoid indole alkaloid dimers	*Melodinus axillari*s (Apocynaceae)	2023	China	CRC (HCT116)	[15]
Axidimin B (**2**)	Monoterpenoid indole alkaloid dimers	*Melodinus axillari*s (Apocynaceae)	2023	China	CRC (HCT116)	[15]
Axidimin C (**3**)	Monoterpenoid indole alkaloid dimers	*Melodinus axillari*s (Apocynaceae)	2023	China	CRC (HCT116)	[15]
Axidimin D (**4**)	Monoterpenoid indole alkaloid dimers	*Melodinus axillari*s (Apocynaceae)	2023	China	CRC (HCT116)	[15]
**(b)**						
**Alkaloids** **(Number)**	**Class**	**Plant Source (Family)**	**Year**	**Country**	**Type of Cancer Cell Line (IC_50_ Values)**	**Reference (Name of First Author)**
Tabersonine (**5**)	Monoterpenoid indole alkaloid	*Melodinus axillari*s (Apocynaceae)	2023	China	CRC (HCT116)	[15]
19*R*-Hydroxytabersonine (**6**)	Monoterpenoid indole alkaloid	*Melodinus axillari*s (Apocynaceae)	2023	China	CRC (HCT116)	[15]
11-Hydroxytabersonine (**7**)	Monoterpenoid indole alkaloid	*Melodinus axillari*s (Apocynaceae)	2023	China	CRC (HCT116)	[15]
11-Methoxytabersonine (**8**)	Monoterpenoid indole alkaloid	*Melodinus axillari*s (Apocynaceae)	2023	China	CRC (HCT116)	[15]
Vandrikidine (**9**)	Monoterpenoid indole alkaloid	*Melodinus axillari*s (Apocynaceae)	2023	China	CRC (HCT116)	[15]
Fusiformine A (**10**)	Monoterpenoid indole alkaloid	*Melodinus axillari*s (Apocynaceae)	2023	China	CRC (HCT116)	[15]
3-oxotabersonine (**11**)	Monoterpenoid indole alkaloid	*Melodinus axillari*s (Apocynaceae)	2023	China	CRC (HCT116)	[15]
3-oxo-11-methoxytabersonine (**12**)	Monoterpenoid indole alkaloid	*Melodinus axillari*s (Apocynaceae)	2023	China	CRC (HCT116)	[15]
Melodinine W2 (**13**)	Monoterpenoid indole alkaloid	*Melodinus axillari*s (Apocynaceae)	2023	China	CRC (HCT116)	[15]
Venalstonidine (**14**)	Monoterpenoid indole alkaloid	*Melodinus axillari*s (Apocynaceae)	2023	China	CRC (HCT116)	[15]
Scandine (**15**)	Monoterpenoid indole alkaloid	*Melodinus axillari*s (Apocynaceae)	2023	China	CRC (HCT116)	[15]
(–)-larutienine A (**16**)	Monoterpenoid indole alkaloid	*Melodinus axillari*s (Apocynaceae)	2023	China	CRC (HCT116)	[15]
Solasonin (**17**)	Steroidal alkaloid	*Solanum nigrum* L. (Solanaceae)	2023	China	CRC (SW620, SW480 and MGC803)	[16]
Berbamine dihydrochloride (**18**)	Bisbenzylisoquinoline alkaloid	*Berberis amurensis* (Berberidaceae)	2023	China	CRC (HT-29, HCT116, RKO and SW480)	[17]
Nitidine chloride (**19**)	Benzophenanthridine alkaloid	*Zanthoxylum nitidum* (Roxb.) DC (Rutaceae)	2022	China	CRC (RKO, HCT116 and HT29)	[18]
GB7 acetate (**20**)	Galbulimima alkaloid	*Galbulimima belgraveana* (Himantandraceae)	2022	China	CRC (HCT116)	[19]
Berberine (**21**)	Protoberberine alkaloid	*Berberis* spp. (Berberidaceae) *Tinospora* spp. (Menispermaceae)	2021	Malaysia	CRC (HCT116)	[21]
		*Coptidis rhizoma* (Ranunculaceae)	2020	China	CRC (HCT116 and SW480, in vitro)	[20]
Boldine (**22**)	Aporphine alkaloid	*Peumus boldus* (Monimiaceae)	2021	Malaysia	CRC (HCT116)	[21]
Worenine (**23**)	Isoquinoline alkaloid	*Coptis chinensis* (Renonculacées)	2021	China	CRC (HCT116 and SW620)	[22]
Chaetocochin J (**24**)	Epipolythiodioxopiperazine alkaloid	*Chaetomium* sp. (Chaetomiaceae)	2021	China	CRC (RKO, HCT116 and SW480)	[23]

## Data Availability

Data are contained within the article.
